# Determination of Bendamustine in Human Plasma and Urine by LC-FL Methods: Application in a Drug Monitoring

**DOI:** 10.1007/s10337-016-3103-3

**Published:** 2016-05-18

**Authors:** Alina Plenis, Agnieszka Frolow, Natalia Rekowska, Ilona Olędzka, Piotr Kowalski, Ewa Bień, Małgorzata Anna Krawczyk, Elżbieta Adamkiewicz-Drożynska, Tomasz Bączek

**Affiliations:** Department of Pharmaceutical Chemistry, Medical University of Gdansk, Hallera 107, 80-416 Gdansk, Poland; Department of Pediatris, Hematology and Oncology; Medical University Gdansk, Debinki 7, 80-11 Gdansk, Poland

**Keywords:** Bendamustine, Biological samples, Sample preparation, LC determination, Fluorescence detection, Drug monitoring

## Abstract

Simple and sensitive liquid chromatography (LC) methods with fluorescence (FL) detection for the determination of bendamustine (BM) in human plasma and urine were developed and validated. The procedure of BM extraction from a plasma sample involved solid-phase extraction with a C18 SPE column, while liquid–liquid extraction with dichloromethane was applied for a urine sample. In both methods, cinoxacin was used as the internal standard. Chromatographic separations were performed on a Synergi Max-RP column, while FL detector was set at the excitation wavelength of 328 nm and the emission wavelength of 420 nm. The LC-FL methods were validated for accuracy, precision, selectivity, linearity, recovery, and stability. The detection limits for BM were 0.5 and 2.5 ng mL^−1^ in plasma and urine, respectively. The intra-day and inter-day precisions were less than 9.86 %, while the accuracies were higher than 92.63 and 94.29 % for BM in plasma and urine, respectively. The proposed LC-FL methods were sensitive, robust, and specific, allowing reliable drug quantification in plasma and urine samples. The methodologies were successfully applied to monitoring of BM in a child with cancer treated with BM.

**Electronic supplementary material:**

The online version of this article (doi:10.1007/s10337-016-3103-3) contains supplementary material, which is available to authorized users.

## Introduction

**Fig. 1:**
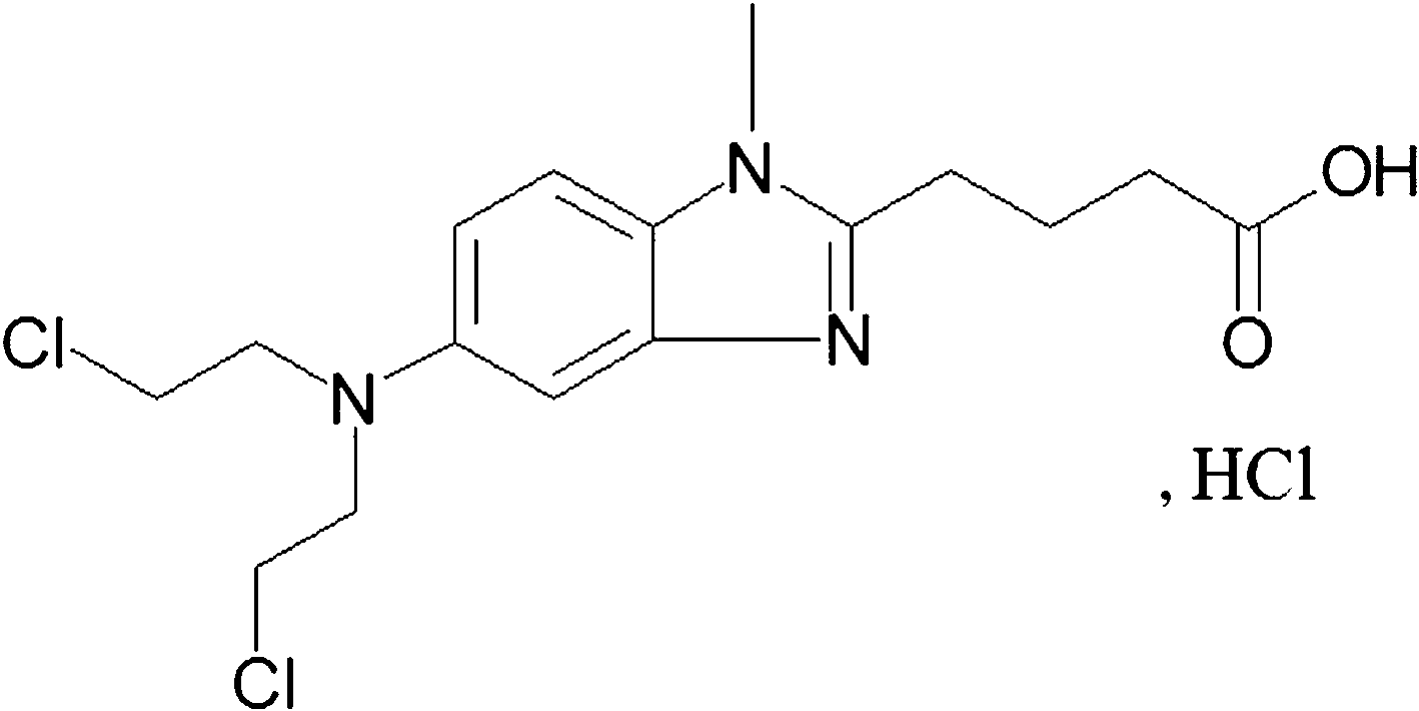
Chemical structure of bendamustine hydrochloride (BM)

Bendamustine (bendamustine hydrochloride) (BM) (4-[5-[bis-(2-chloroethyl)amino]-1-methyl-benzimidazol-2-yl]butyric acid hydrochloride is a unique alkylating agent, which combines a nitrogen mustard moiety of mechlorethamine with a benzimidazole (Fig. 1) [1]. This cytostatic agent has been used in Germany since the 1970s against a number of malignancies [2, 3]. In 2008, BM was approved in the US for the treatment of chronic lymphocytic leukemia (CLL) and later for indolent B cell non-Hodgkin’s lymphoma (NHL) [4, 5]. The mechanism of action of BM is unique and multifaceted. Compared to other alkylating agents, BM causes more extensive and long-lasting DNA damage which leads to a concentration-dependent apoptosis and non-apoptotic cell death or mitotic catastrophe [6–8]. Thus, many clinical trials have recently focused on the assessment of the optimal dosage, tolerance, and efficacy of BM in various hematological and some solid malignancies, including: CLL, NHL, Hodgkin’s disease, multiple myeloma, primary and metastatic brain tumors, small-cell lung cancer sarcomas, and other neoplasms [9–14]. Preclinical and clinical studies have shown that BM demonstrates no complete cross-resistance with the conventional alkylating agents, which may explain its efficacy in heavily pretreated, relapsed, and/or progressive malignancies [15].

BM can be used as a single agent or in combination with other anti-cancer agents [16, 17]. Although various BM dosing schemes have been attempted, the most typical protocol consists in administering BM intravenously on two consecutive days, every 28 days, in the dose of 100–120 mg (m^2^)^−1^ body surface per day.

The most common side effects associated with BM include hematological events, such as leukopenia, neutropenia, thrombocytopenia, and anemia and gastrointestinal complications [18, 19]. It should be noted that the major route of BM metabolism is its hydrolysis to inactive products which add little or nothing to the anti-cancer effects of BM [16]. Thus, clinical activity against a variety of hematological malignancies and solid tumors is mainly related to the concentration of the parent substance, which indicates that its dosage should be carefully adjusted for the treatment of patients with a particular type of cancer [20–23]. Moreover, clinical trials on the use of BM in cancer children are seldom described, which increase the risk of incorrect drug administration [24, 25]. Therefore, it is important to monitor the BM concentration in biological fluids to optimize efficacy and minimize any adverse effects [26].

To the best of our knowledge, only a few analytical techniques have been developed for the quantification of BM in the presence of its metabolites in plasma [23, 27–29], urine [23, 27–31], bile [23, 31, 32], or mouse brain tissue [33]. Among them, liquid chromatography (LC) coupled with mass spectrometry (MS) has been the most widely used technique [22, 23, 27, 29–33]. Unfortunately, these methodologies are scarred with a number of limitations, such as long analysis time and no validation data [22, 23, 27, 30–32], 100-fold dilution of the urine sample with plasma [29], and low recovery of BM from brain tissue [33]. In addition, LC methods with fluorescence (FL) detection have been reported for the monitoring of BM in biological fluids in the presence of its metabolites [22, 23, 28, 31, 32], respectively. However, those LC-FL techniques suffer from insufficient validation information [22, 23, 31, 32] and low efficiency of the extraction procedure [28].

The aim of this study was to develop rapid, cost-effective, and sensitive LC methods with FL detection, in combination with simple sample preparation procedures suitable for the quantification of BM in human plasma and urine, which could be accessible to most pharmaceutical and clinical laboratories offering interesting alternatives to the previously published methodologies. The utility of the proposed LC-FL methods was demonstrated through their application to monitor BM in a pediatric cancer patient.

## Materials and Methods

### Reagents

Bendamustine hydrochloride (99.8 % purity) was supplied by Tocris (Bristol, United Kingdom). Cinoxacin (internal standard, I.S.) (99.8 % purity) came from Sigma Aldrich (St. Louis, MO, USA). *Ortho*-phosphoric acid 85 % and hydrochloric acid 36 % of the analytical grade were purchased from POCh (Gliwice, Poland). HPLC grade acetonitrile, methanol, and dichloromethane were supplied by Merck (Darmstadt, Germany). The organic solvents and reagents used for sample preparation and the mobile phase were used as received without further purification. The water used in all experiments was purified with the Milli-Q system (Molsheim, France). LiChrolut RP-18 cartridges (100 mg, 1 mL) were purchased from Merck (Darmstadt, Germany). The control plasma and urine were obtained from healthy volunteers.

### Chromatographic Conditions

All LC measurements were performed on an ACME 9000 system (Younglin Instrument Corporation, Anyang, The Republic of Korea) equipped with a pump (SP 930D), fluorescence detector RF-551 (Shimadzu, Japan), autosampler, thermostat (CTS30), and computer system for data acquisition (AutoChro-3000). Chromatographic separation was carried out on a Synergi Max-RP column (150 × 4.6 mm, 4 μm) from Phenomenex (Torrance, CA, USA) with a binary mixture of acetonitrile–water (25:75, v/v) adjusted to pH 2.7 with 85 % *ortho*-phosphoric acid, used as the mobile phase. The flow-rate was 1 mL min^−1^, whereas the column temperature was 30 °C. The analytes were monitored with an FL detector set at the excitation wavelength of 328 nm and the emission wavelength of 420 nm, respectively. A sample volume of 10 μL was injected into HPLC system.

### Standard Solutions

The stock standard solutions of BM and the I.S. (1 mg mL^−1^) were prepared by weighing accurately 10.0 mg of the compound of interest and diluting it in 10 mL of methanol. Standard working solutions of BM were prepared daily by diluting the stock solution appropriately with methanol, so as to obtain the concentrations of 100, 10, 1 μg mL^−1^, and 100 ng mL^−1^. The internal standard stock solution was diluted further, also with methanol, to prepare the working standard solution containing 10 μg mL^−1^ of cinoxacin. The stock and the working standard solutions of BM were stored at −80 °C, while the stock standard solution of the I.S. and its working solution were stored in a refrigerator protected from light.

### Plasma and Urine Standards

The calibration samples (CS) and quality control samples (QCs) were prepared in drug-free plasma (100 µL) and drug-free urine (500 µL) as fortified samples. The calibration plasma samples were prepared by spiking with the appropriate working standard solutions of BM to obtain ten concentration levels of 1, 25, 100, 250, 500, 1000, 2000, 4000, 6000, and 8000 ng mL^−1^, and the I.S. concentration of 1500 ng mL^−1^. The plasma QCs were prepared with three BM levels (25, 500 and 6000 ng mL^−1^) at the I.S. concentration of 1500 ng mL^−1^, respectively.

To minimize the degradation of BM in urine, CS and QCs were stabilized by adding 100 μL of 6 M HCl to 5 mL of urine prior BM spiking into the sample. In this final biological matrix, a set of calibration standards with BM levels of 5, 50, 250, 500, 1000, and 2000 ng mL^−1^ at the I.S. concentration of 1500 ng mL^−1^ was prepared. QC samples were prepared by spiking the acid-treated human urine samples with BM to produce the concentration pools of 50, 250, and 500 ng mL^−1^ at the I.S. level of 1500 ng mL^−1^.

### Sample Preparation

Quality control plasma samples, calibration standard plasma samples, control blank plasma, and real clinical plasma samples were analyzed in the same manner. Thus, human plasma volumes, 100 µL each, were transferred to a polypropylene centrifuge tube, and the I.S. solution at the concentration of 1500 ng mL^−1^ and different concentrations (1–8000 ng mL^−1^) of BM were added, then vortex-mixed for 30 s. Next, 1 mL of 0.1-M hydrochloric acid was added to the plasma sample, which was then vortex-mixed for 30 s and shaken mechanically for 10 min. After centrifugation for 5 min at 4 °C (8000*g*), solid-phase extraction (SPE) was performed on a LiChrolut RP-18 cartridge conditioned with 1 mL of methanol and 1 mL of water before the sample loading. Next, the cartridge was washed with 1 mL of 5 % methanol, and vacuum dried for 1 min. Then, the compounds of interest were eluted with 1 mL of methanol. The whole solvent was evaporated to dryness at 50 °C under vacuum. Finally, the residue was reconstituted with 50 µL of methanol, vortex-mixed for 30 s, and lastly added 100 μL of the mobile phase. After centrifugation at 8000*g* for 5 min, the supernatant was transferred to a dark-colored autosampler vial, which then was stored at 2–8 °C pending analysis. Finally, a 10-μL aliquot sample was injected into the LC system.

Just as above, the quality control urine samples, calibration standard urine samples, control blank urine, and real clinical urine samples were processed identically, and the final extracts were used to quantify BM. Human urine volumes, 500 µL each, were transferred into a polypropylene centrifuge tube and vortex-mixed with 20 μL of 6-M hydrochloric acid. Next, the I.S. solution at the concentration level of 1500 ng mL^−1^ and different concentrations of BM, ranging between 5 and 2000 ng mL^−1^ were added. After vortex-mixing for 30 s, liquid–liquid extraction (LLE) was performed using 2 mL of dichloromethane. The content of the tube was vortex-mixed for 30 s, shaken mechanically for 10 min, and after centrifugation for 5 min at 4000*g*, the dichloromethane layer was quantitatively transferred into another clean glass test tube and evaporated to dryness at 45 °C under vacuum. The residue was reconstituted in 50 μL of methanol and vortex-mixed for 30 s, then followed by addition of 50 μL of the mobile phase. After centrifugation at 8000*g* for 5 min, the supernatant was transferred to a dark-colored autosampler vial, which then was stored at 2–8 °C pending analysis. Finally, a 10-μL aliquot sample was injected into the LC system.

### Validation of Analytical Methods

The LC methods were fully validated according to the requirements of the Food and Drug Administration (FDA) and International Conference on Harmonization of Technical Requirements for Registration of Pharmaceuticals for Human Use (ICH) with emphasis laid on selectivity, linearity, sensitivity, accuracy and precision, and extraction recovery and stability [34, 35]. This assay was performed by assaying six replicates of the calibration standards (CS) and six replications of the quality control (QC) samples prepared in three (low, middle, and high) concentration levels in human plasma and urine, respectively. In both the cases, calibrations were performed using the least-squares linear regression model in the form of *y* = a + b*x*, where *y* is the peak area ratio of BM to the I.S. and *x* represents the respective standard plasma/urinary concentration of BM. To evaluate linearity, pooled plasma was spiked with the working standard solutions of BM to obtain concentrations in the range of 1–8000 ng mL^−1^ at the I.S. level of 1500 ng mL^−1^, respectively. As for the urine samples, these were spiked with BM in the range of 5–2000 ng mL^−1^ at the I.S. concentration of 1500 ng mL^−1^, respectively. Both plasma and urine calibration samples were analyzed on the same day.

The limit of detection (LOD) was measured as the sample concentration for which the area peak was three times that of the baseline noise. The lower limit of quantification (LLOQ) was defined as the lowest concentration for which the ratio of the signal-to-noise was higher than 10, and which could be analyzed with the accuracy of ±20 % and precision ≤15 %.

Selectivity of the method was determined by an LC analysis of six blank plasma and six urine samples of different origins for peak interference in the BM and I.S. retention times.

To assess the intra- and inter-day precision and accuracy, parallel analytical runs were performed on the same day, and on 6 days over 2 months, respectively. Therefore, intra-day precision and accuracy were measured by the six-fold replicate analysis of CS at each concentration used to prepare the calibration plots in plasma and urine, respectively. Inter-day precision and accuracy were determined by an analysis of QC samples: 25, 500, and 6000 ng mL^−1^ for BM in plasma, and 50, 250, and 500 ng mL^−1^ for BM in urine, respectively. The I.S. concentration in the plasma and urine QC samples was identified as 1500 ng mL^−1^. Precision was expressed as the relative standard deviation (RSD), whereas accuracy was established as the percentage difference between the measured concentration and the nominal concentration.

The absolute extraction recoveries of BM from plasma and urine were determined by comparing the peak areas of the compounds of interest in the extracted plasma and urine samples with the non-extracted ones at equivalent concentrations (100 and 1000 ng mL^−1^ for BM, respectively). Each sample was analyzed in triplicate. The absolute recovery of the I.S. in the plasma and urine samples was determined for the concentration of 1500 ng mL^−1^ (*n* = 6).

Several stability tests were performed as part of validation under various conditions, including short-term stability (at room temperature for 4 h), long-term stability (frozen at −80 °C for 3 months), freeze/thaw stability (three cycles from −80 °C to room temperature), and post-preparative storage (at 4 °C for 24 h), for fortified plasma and urine samples at low, medium, and high QC concentrations of BM. In each test stability assay, three replicates were analyzed using the proposed LC methods.

### Method Application

The LC method was used to determine the levels of BM in human plasma and urine after intravenous infusion of the compound to a child with refractory and progressive Hodgkin’s lymphoma for two consecutive days. The boy’s age was 17, and his weight was 50 kg. The condition of the patient was established through medical history, and clinical and laboratory examinations. Both the parents and the patient read the protocol and gave their written informed consent before joining the study. The clinical protocol was approved by the Ethical Committee of the Medical University of Gdańsk (Gdańsk, Poland). BM was administered in monotherapy as a 60-min intravenous (IV) infusion at the dose of 120 mg (m^2^)^−1^ body surface per day, for two consecutive days.

Venous blood samples (4 mL each) were collected in K_2_EDTA tubes at 0 (pre-dose baseline), 0.5, and 1.0 h after the initiation of the first infusion, and at 1, 2, 3, 4, 6, 8, 12, 18, and 23 h after the end of the first infusion. Then, blood sample collection continued over the second infusion (0.5 and 1 h), and after 1, 2, 3, 4, 6, 8, 12, 18, 24, 48, and 72 h following the completion of the second BM infusion. All plasma samples were immediately separated by centrifugation at 3000*g* for 10 min, and the obtained plasma was stored at −80 °C up to the LC analysis.

Urine samples were collected after the first IV infusion of BM (12.00 a.m.), at 14.00; 17.25; 9.50, as well as before the second IV infusion (11.50) (1st day); and after the second IV infusion (12.00 a.m.) at 14.00; 17.00; 21.40; 9.40 (2nd day), as well as at 12.00 (3rd day), and at 12.00 (4th day) after the end of drug infusion. Moreover, 24-h urine samples after day 1 and day 2 of BM administration were collected. To prevent chemical hydrolysis of BM, each urine sample (5 mL) was immediately frozen and stored at −80 °C in a polypropylene tube previously treated with 100 μL of 6 M HCl.

### Results and Discussion

In view of pharmacokinetic and biomedical studies, the aim of this study was to develop the sensitive LC methods with fluorescence detection for the quantification of BM in human plasma and urine to offer an interesting alternative to the LC methodologies previously reported in the literature [22, 23, 27–33]. Thus, a lot of experiments were conducted to identify the optimal conditions of sample collection and storage, to develop a simple sample preparation procedure, and to establish the chromatographic parameters for the LC analysis of BM in biological fluids.

### Method Development

#### Sample Collection and Storage

The literature data on stability of nitrogen mustard-containing compounds, such as BM, indicate that the substances are unstable in aqueous solutions because of degradation by hydrolysis [36–38]. Moreover, the process is strictly pH dependent. For example, the BM loss was higher at pH 7.6 than the loss levels observed at pH 6.5, with the lowest BM degradation found at a pH < 3 [29, 37]. Therefore, following the literature data, various options for storing BM urine samples were investigated, including the addition of 6-M HCl-NaCl (1–10 mL urine) [22, 23, 28, 32], and 100-fold dilution in human plasma which enabled processing and analyzing the urine-plasma samples in the same manner as the human plasma samples [29]. On the other hand, this step might also decrease the reliability of BM quantifications in urine because of a significant dilution of the real clinical sample.

In the presented study, 100 μL of 6-M HCl was added to each tube prior urine sample loading (5 mL) to prevent chemical hydrolysis of BM. The samples were immediately frozen and stored at −80 °C up to the LC analysis. This procedure can be deemed an interesting alternative to using 6-M HCl-NaCl, because the lesser degradation of the biological matrix, combined with decreased dilution of the urine sample (1:10 vs 1:50, v/v), enables improving sensitivity and reliability of the BM assay in urine samples.

#### Optimization of Sample Preparation

When developing the sample preparation procedure, various organic solvents, such as *tert*-butyl methyl ether (TBME), ethyl acetate, and dichloromethane were used for liquid–liquid extraction (LLE) of BM from human plasma samples. Moreover, solid-phase extraction (SPE) procedures using C18, C8, and hydrophilic-lipophilic balance (HLB) cartridges, and three solvents for the elution of the compounds of interest (acetonitrile, methanol, and dichloromethane) were tested. The efficiency of each studied procedure was determined on the basis of the LC analysis of plasma samples spiked with BM at the levels of 100 and 1000 ng mL^−1^, then compared to the absolute recovery data calculated for other procedures studied. Moreover, signal-to-noise ratios were calculated, expressed as: the area peak of the analyte in the spiked biological sample, and the signal area for the same retention time in the blank biological sample. The assay was performed to evaluate the clean-up of the matrix background. The obtained mean recovery data (mean ± SD) for BM from plasma samples are shown in Table S1 (see Electronic Supplementary Material). The results visualize that the highest efficiency of the tested LLE in plasma samples was found when dichloromethane was used as the extraction solvent (76.6 ± 5.4 %), but the BM recovery ratio was lower than for the SPE procedures. Among the latter, the SPE methods based on a LiChrolut RP-18 cartridge were the most efficient. It was also observed that the best elimination of the ballast substances from the plasma matrix was achieved for dichloromethane (data not shown); however, the disadvantage was lower recovery of BM (94.0 ± 5.4 %) than after the use of methanol (99.8 ± 3.5 %). Thus, methanol was finally selected for further studies because of higher clean-up of the plasma background and the highest recovery of BM. In those SPE conditions, the rate of recovery for the I.S. was 97.5 ± 3.8 %.

When optimizing the sample preparation procedure for urine samples, 20 μL of 6-M HCl was added to thawed urine (0.5 mL) to achieve a low pH biological matrix, and thus minimize BM degradation. Next, all of the above-described sample preparation procedures were also used. Unfortunately, the application of all studied SPE procedures brought unsatisfactory results irrespective of the urine volume loaded to the SPE cartridges (100–500 μL). That was due to insufficient clean-up of the urine background from the ballast substances in the retention times of both the BM and the I.S. (data not shown). Out of the tested LLE procedures, the highest obtainable clean urine extract combined with high recovery of BM was found when dichloromethane was used (Electronic Supplementary Material see Table S1). In the proposed LLE method, the rate of recovery for the I.S. (cinoxacin) was 98.5 ± 6.5 %.

These data evidence that the efficiency of BM extraction from plasma based on the developed SPE with C18 cartridges was higher than that of using protein precipitation with 10 % perchloric acid/methanol solution (68.7–72.2 % for BM) [28], or SPE with HLB Oasis columns (76.4–82.8 % for BM) [29], or protein precipitation with methanol for brain tissues (41.1–69.2 % for BM) [33]. Noteworthy, the rate of recovery of BM from urine based on LLE with dichloromethane was also higher than the rates of the methods earlier reported in the literature [29, 33]. Thus, both SPE with C18 and LLE using dichloromethane can be considered as interesting alternatives to the previous published sample preparation procedures.

#### Optimization of LC Parameters

For the optimization of the LC conditions for BM determination, several analytical columns like Synergi Polar-RP, Discovery HS C18, Synergi Hydro-RP, InertSustain C18, and Synergi Max-RP were tested and compared for their signal response to BM, the peak shape, and the retention times. Finally, the Synergi Max-RP (4.6 mm × 150 mm, 4 µm) column was chosen to quantify BM in human plasma and urine, because of its highest signal response to BM, its symmetric peak shape, and the acceptable retention time. This column, but having different dimensions, was also used for the quantification of BM in the presence of its metabolites described by Teichert et al. [22, 23, 32].

When the mobile phase composition was optimized, various mixtures of acetonitrile and/or methanol with water were prepared at different proportions, following which the pH of those mobiles phases was adjusted with 85 % *ortho*-phosphoric acid to 2.5, 2.7, 2.9, 3.1, 3.3, and 3.5. The mixture of acetonitrile and water in the ratio of 25:75 (v/v, pH 2.7) was chosen as optimal for the LC separation of BM, since it ensures good peak separation and appropriate retention times. It should also be noted that the acidic pH of the mobile phase in the LC separation was able to prevent chemical hydrolysis of BM, whereas the isocratic elution of the mobile phase can considered as an interesting alternative to earlier described gradient mode LC methods for BM quantification in biological samples [22, 23, 27, 29–33].

Other experimental parameters, such as the column temperature and the flow-rate of the mobile phase, were also tested. Thus, increasing the column temperature and the flow-rate of the mobile phase caused shortened the retention times of the BM and the I.S., but the signal peaks of the analytes interfered with the peaks of the endogenous matrix substances. In effect, the LC separation at 30 °C and the flow-rate of 1 mL min^−1^ were chosen as a good compromise between shorter retention times and the best resolution.

With the view of optimizing the fluorescence detection conditions, different excitation wavelengths (315–340 nm) and emission wavelengths (410–435 nm) were tested. The highest response signals for BM were observed at the excitation wavelength of 328 nm and emission wavelength of 420 nm, respectively. Hence, these parameters of FL detection were finally selected for further study. Those results corroborated the data previously reported in the literature [22, 23, 28, 32].

The developed isocratic mode LC conditions for the quantification of BM in biological fluids offer satisfactory separation of BM at a shorter analysis run time (10 min) compared to the previously reported LC-FL techniques—70 min [22, 23, 32] and 14 min [28], respectively. This analysis time is comparable to the time obtained for the gradient mode LC–MS/MS, 11 min [29] and 8.2 min [33], but significant shorter than required for the LC–MS/MS method with off-line radioactivity detection (180 min) [30], respectively. In effect, the proposed LC-FL methods can be considered as interesting alternatives for most pharmaceutical and clinical laboratories.

#### Validation of Analytical Methods

For method validation purposes, the calibration samples (CS) and quality control samples (QCs) were prepared as described in “Plasma and Urine Standards”, and treated in the same manner as reported in “Sample Preparation”, then analyzed in the LC conditions described in “Chromatographic Conditions”. One of the important aspects of the validation of the method was the choice of the internal standard. For this propose, cinoxacin was selected, because this drug offers good solubility in the most organic solvents and possess natural fluorescence activity in the range required for BM. In addition, the retention time of cinoxacin was optimal in chromatographic conditions used for BM assay. Moreover, this substance is not used in human medicine.

### Selectivity

**Fig. 2:**
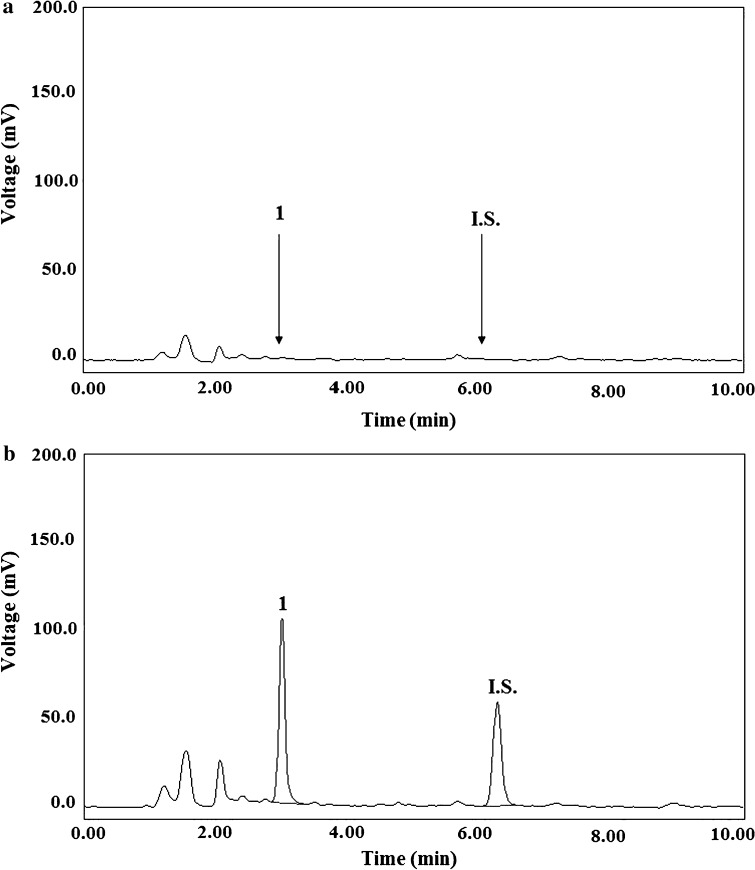
Typical chromatograms of the blank human plasma extract (**a**) and of a plasma sample spiked with BM (1) (500 ng mL^−1^) and cinoxacin (I.S.) at the level of 1500 ng mL^−1^ (**b**) after the SPE using the C18 cartridges

**Fig. 3:**
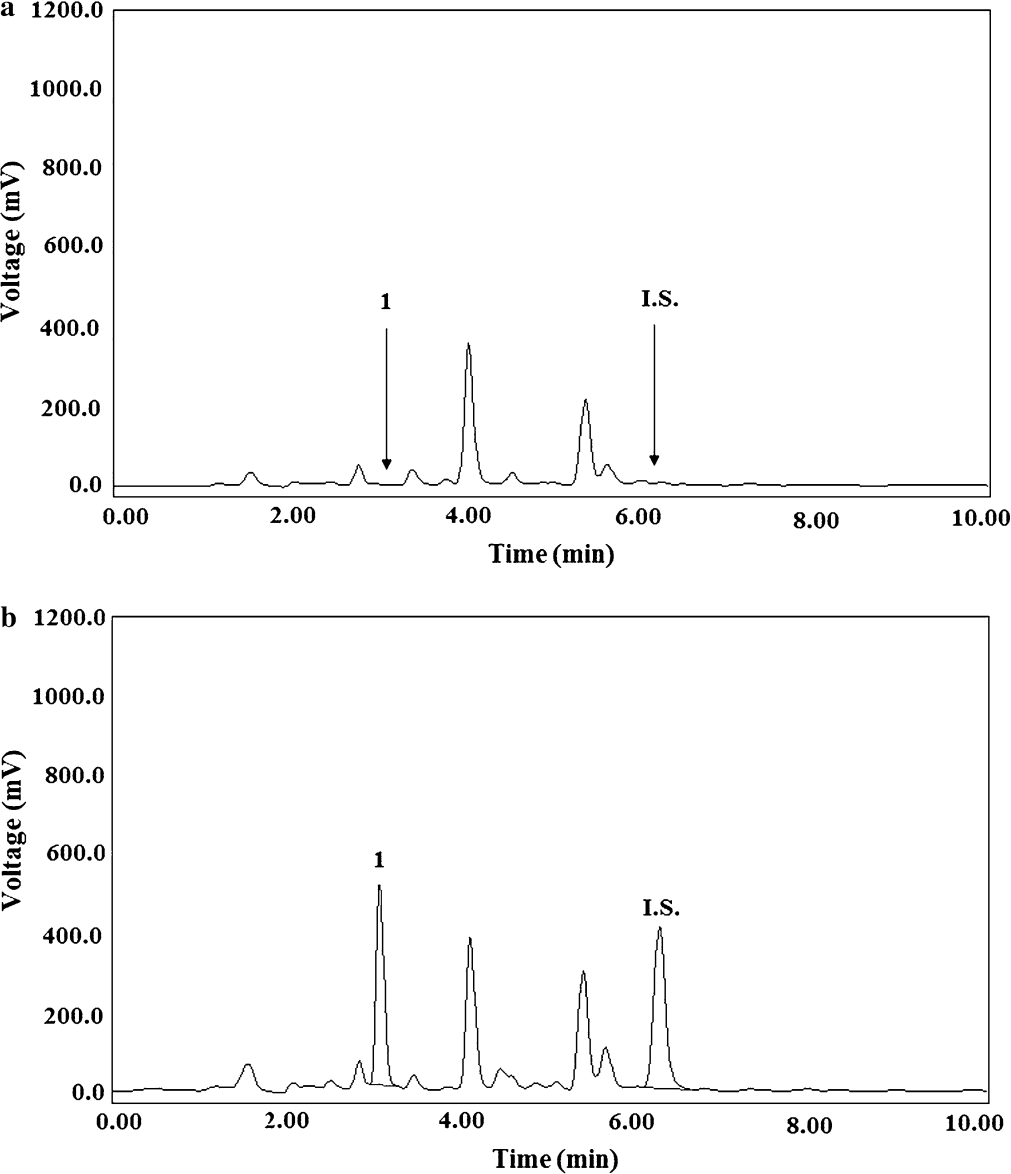
Typical chromatograms of the blank human urine extract (**a**) and of a urine sample spiked with BM (1) (500 ng mL^−1^) and cinoxacin (I.S.) at the level of 1500 ng mL^−1^ (**b**) after the LLE with dichloromethane

Selectivity was evaluated by comparing chromatograms of drug-free blank human plasma and those of urine samples and the ones of extracts from plasma and urine containing BM and the I.S., respectively, all tested for interference using the proposed LC methods (*n* = 6). Representative chromatograms of drug-free plasma and plasma spiked with 500 ng mL^−1^ of BM and with the I.S. at the level of 1500 ng mL^−1^ are shown in Fig. 2a and b, respectively. Typical chromatograms of drug-free urine and urine spiked with 500 ng mL^−1^ of BM and with the I.S. at the concentration of 1500 ng mL^−1^ are presented in Fig. 3a and b, respectively. The chromatograms confirm that no interferences were detected from the substances of the endogenous matrix in the area, where BM and the I.S. appear.

### Linearity

The linearity of the method was confirmed over the concentration ranges of 1–8000 and 5–2000 ng mL^−1^ for BM in plasma and urine, respectively. The corresponding calibration parameters of both calibration curves are summarized in Table 1. The data confirm that the calibration curves demonstrated good linearity for both plasma and urine, with the correlation coefficients (*R*^2^) higher than 0.9996, which indicate excellent linearity of the methods within the considered linear ranges.

**Table 1:** Summary of validation data for BM determination in human plasma and urine by the LC-FL methods (*n* = 6)

	Plasma	Urine
Linearity (ng mL^−1^)	1–8000	5–2000
Equation parameter		
Slope	0.0040 ± 0.00002	0.0021 ± 0.00002
Intercept	0.0127 ± 0.0620	−0.0043 ± 0.0201
Standard error	0.152	0.0363
Correlation coefficient (*R* ^2^)	0.9998	0.9996
LOD (ng mL^−1^)	0.5	2.5
LLOQ (ng mL^−1^)	1.0	5.0

### Limits of Detection and Quantification

The limits of detection (LOD) were measured for six independently made replications and were found to be 0.5 and 2.5 ng mL^−1^ for BM in plasma and urine, respectively. The lower limits of quantification (LLOQ) for BM were estimated by the six-fold replicate analysis, and found to stay at the level of 1 and 5 ng mL^−1^ in plasma and urine samples, respectively. The LLOQ for BM were also the lowest standard concentrations in both calibration curves (Table 1). The LLOQ parameters were lower than those published heretofore for the LC-FL assay of BM in plasma [28], LC–MS/MS determination in urine [27, 29], and mouse brain tissue [33], while being comparable to those obtained by LC-FL for BM in urine [28], even though lower plasma and urine volumes were used (100 vs 200–250 μL for plasma and 500 vs 1000 μL for urine, respectively) [22, 23, 28, 29, 32].

### Precision and Accuracy

The intra-day and inter-day precision and accuracy results for both the LC methods are summarized in Table 2. The data confirmed that intra-day precisions for BM in plasma and urine were below 9.65 and 9.86 %, while intra-day accuracies were higher than 92.63 and 94.29 %, respectively. The inter-day precisions did not exceed 9.82 and 9.02 %. The inter-day accuracies ranged from 100.26 to 101.16 % and between 99.75 and 101.74 %, respectively. Those data confirmed that the intra-day and inter-day accuracy and precision values met the generally accepted criteria for bioanalytical method validation at all calibration and QC concentration levels [34, 35].

**Table 2:** Intra-day and inter-day precision and accuracy for the determination of BM in plasma and urine samples by SPE-LC and LLE-LC techniques, respectively

Intra-day (*n* = 6)				Inter-day (*n* = 6)			
Concentration (ng mL^−1^)		Precision RSD (%)	Accuracy (%)	Concentration (ng mL^−1^)		Precision RSD (%)	Accuracy (%)
Spiked	Found (mean ± SD)			Spiked	Found (mean ± SD)		
Plasma							
1	1.08 ± 0.10	9.65	107.92	25	25.29 ± 2.48	9.82	101.16
25	24.08 ± 2.06	8.54	96.30	500	501.28 ± 5.64	1.12	100.26
100	106.58 ± 7.75	7.27	106.58	6000	6018.21 ± 48.73	0.81	100.30
250	231.58 ± 13.42	5.79	92.63				
500	489.08 ± 23.93	4.89	97.82				
1000	1034.08 ± 38.50	3.72	103.41				
2000	2049.08 ± 41.50	2.03	102.45				
4000	3926.58 ± 62.91	1.60	98.16				
6000	6049.08 ± 66.24	1.10	100.82				
8000	8029.08 ± 43.99	0.55	100.36				
Urine							
5	4.71 ± 0.46	9.86	94.29	50	49.87 ± 4.50	9.02	99.75
50	49.19 ± 4.23	8.59	98.38	250	254.34 ± 10.47	4.12	101.74
250	249.67 ± 15.94	6.38	99.87	500	505.49 ± 3.73	0.74	101.10
500	487.76 ± 19.98	4.10	97.55				
1000	1035.38 ± 24.28	2.35	103.54				
2000	2002.05 ± 31.15	1.56	100.10				

Than 9.86 % while the accuracies were in the range of 92.63–107.92 and 94.29–103.54 %

### Recovery

The extraction recoveries of BM from plasma and urine were measured at two different concentration levels (low and high), six replicates for each concentration. The obtained recovery data for BM are summarized in Table S1 (see Electronic Supplementary Material). The I.S. (cinoxacin) recovery rate from human plasma using SPE-C18 was attained at 97.5 ± 3.8 %, whereas the value of 98.5 ± 6.5 % was calculated for the urine sample after the LLE with dichloromethane. These extraction recovery data confirm that the developed sample preparation procedures can assure adequate sensitivity for processing plasma and urine samples for the LC assay of BM. Moreover, these procedures are more effective than the previously reported methods [28, 29, 33].

### Stability Tests

Stability tests for various conditions, as described in the section “Validation of Analytical Methods”, were conducted by assessing plasma and urine samples at three QC levels of BM. Representative chromatograms of plasma spiked with 500 ng mL^−1^ of BM and with the I.S. at the level of 1500 ng mL^−1^ obtained in short-term stability and long-term stability tests are shown in Fig. S1a and S1b, whereas the chromatograms performed in freeze/thaw stability and post-preparative storage tests are presented in Figure S2c and S2d, respectively (see Electronic Supplementary Material). Typical chromatograms of urine spiked with 250 ng mL^−1^ of BM and with the I.S. at the level of 1500 ng mL^−1^ obtained in four stability tests are illustrated in Figs. S3 and S4, respectively (see Electronic Supplementary Material). All stability evaluations were based on back-calculated concentrations. The resulting data are summarized in Table 3 revealed no significant degradation of BM in the plasma and urine samples. Thus, all samples prove stable and could be handled in normal laboratory conditions without any significant loss, if under the conditions described above.

**Table 3:** Stability of BM in plasma and urine samples (*n* = 3)

Plasma	Concentration (ng mL^−1^)		
	25	500	6000
	Accuracy (mean ± SD) (%)		
Short-time stability	97.7 ± 7.8	101.7 ± 5.5	102.1 ± 6.8
Long-time stability	109.9 ± 6.9	95.9 ± 3.9	98.1 ± 8.9
Freeze/thaw stability	95.7 ± 6.7	102.6 ± 8.1	96.6 ± 7.8
Post-preparative storage	103.9 ± 3.9	98.3 ± 6.9	101.2 ± 7.9

Urine	Concentration (ng mL^−1^)		
	50	250	500
	Accuracy (mean ± SD) (%)		
Short-time stability	103.4 ± 4.9	102.9 ± 7.9	96.6 ± 6.6
Long-time stability	104.3 ± 8.8	94.7 ± 5.5	97.9 ± 4.1
Freeze/thaw stability	93.9 ± 3.9	96.9 ± 3.9	95.9 ± 3.9
Post-preparative storage	99.7 ± 4.4	101.3 ± 6.6	101.7 ± 7.3

#### Method Application to the Real Plasma and Urine Samples

It should be noticed that clinical trials on the application of BM in cancer children are seldom reported which causes higher risk of incorrect drug administration [24, 25]. In effect, the efficacy of the therapy and high level of adverse effects can be observed [26].

**Fig. 4:**
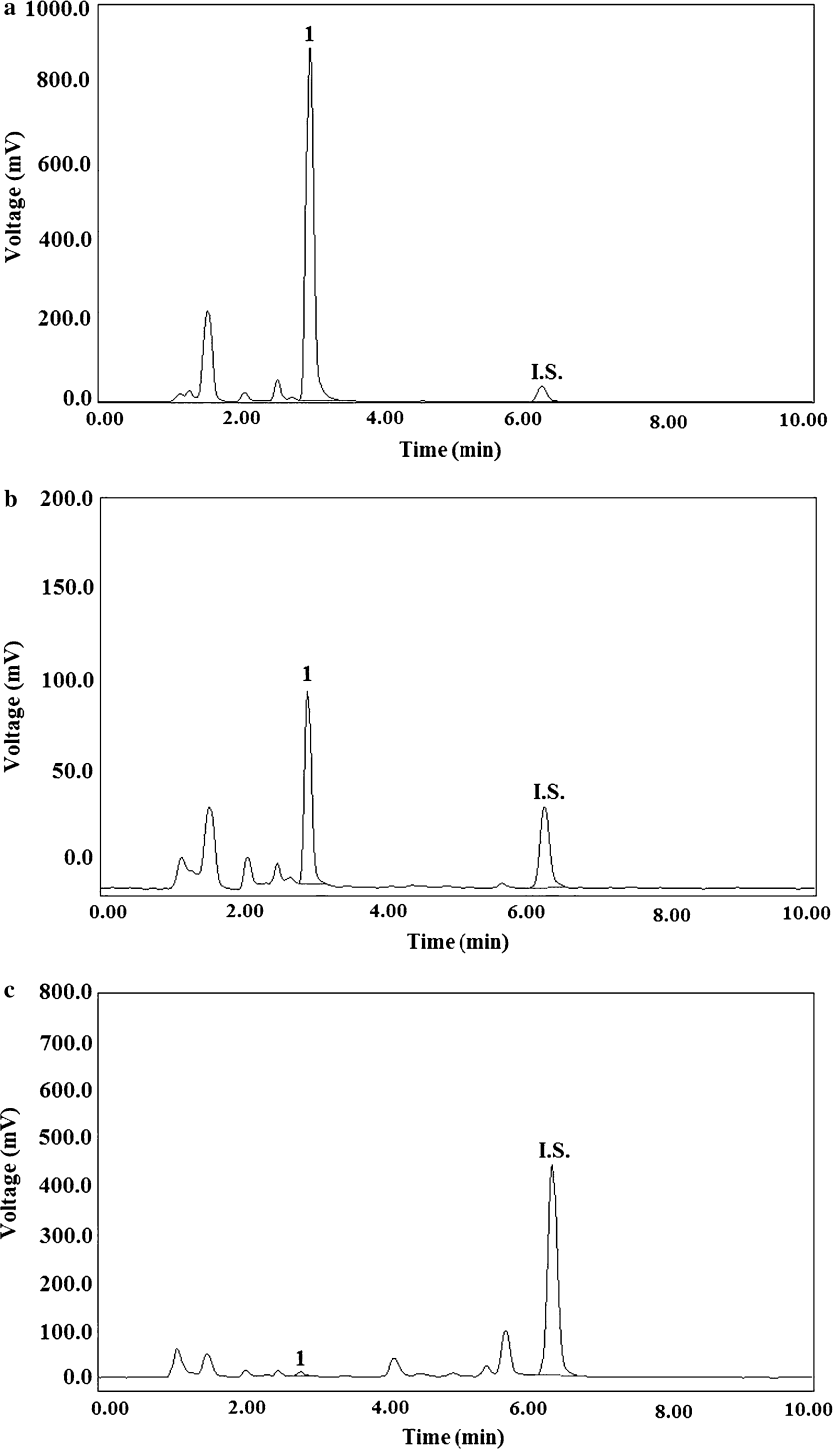
Representative LC chromatograms of human extracts obtained from a 17-year-old patient with refractory progressive Hodgkin’s lymphoma, after IV infusions at the dose of 120 mg (m^2^)^−1^ body surface per day, administered for two consecutive days at the following BM levels (1): **a** 7015.36 ng mL^−1^ in the plasma sample collected after the end of the first IV infusion; **b** 487.37 ng mL^−1^ in the plasma sample collected 1 h after the end of the first IV infusion; and **c** 13.18 ng mL^−1^ in the urine sample collected at 21.40 after the second IV infusion (12.00 a.m.), and cinoxacin (I.S.) at the concentration of 1500 ng mL^−1^

**Fig. 5:**
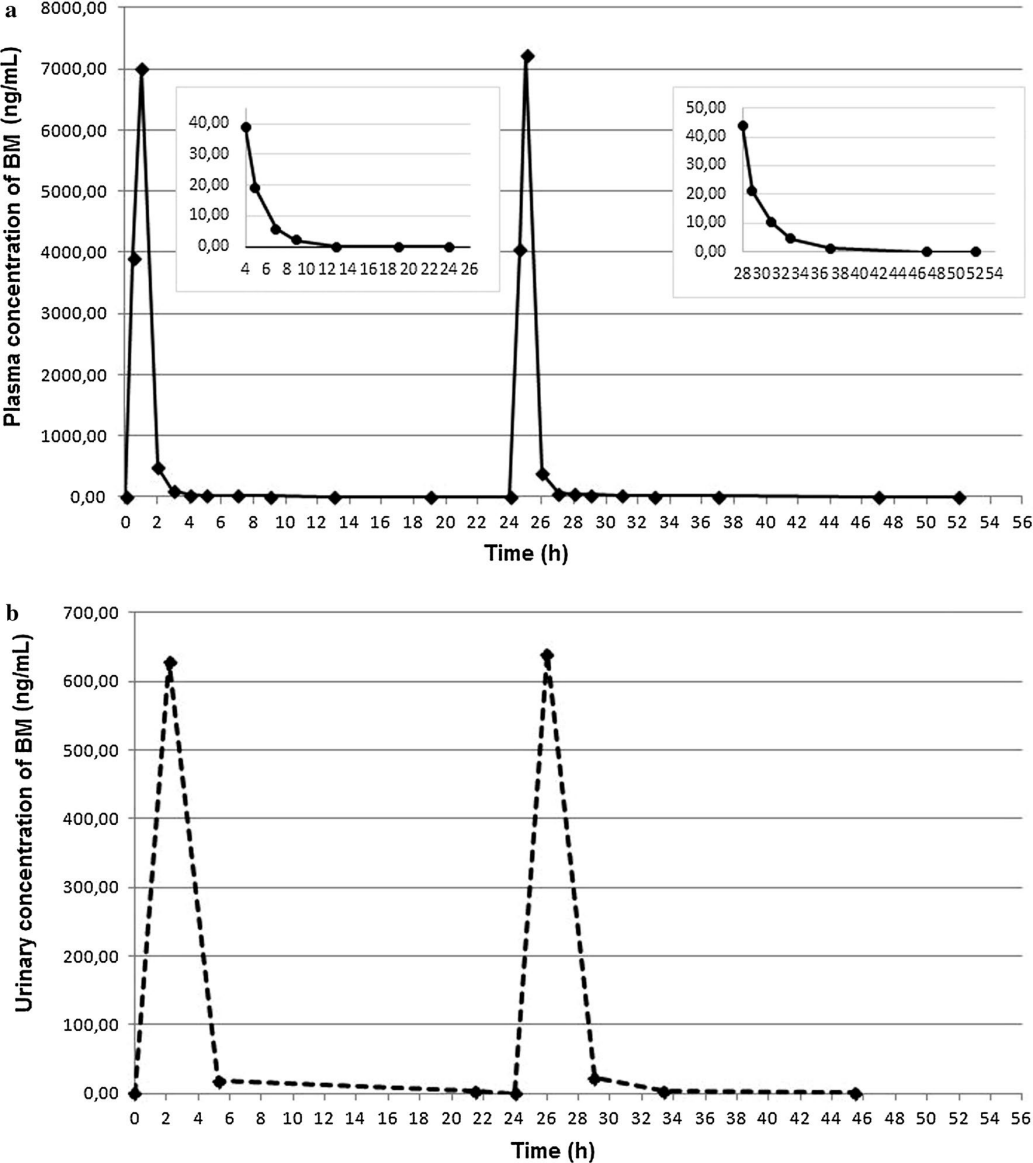
BM concentration profiles in the plasma (**a**) and urine (**b**) of a 17-year-old patient with NHL, after IV infusions at the dose of 120 mg (m^2^)^−1^ body surface per day, administered for two consecutive days

The developed LC methods have been successfully used in monitoring of BM administered to a 17-year-old boy with refractory progressive Hodgkin’s lymphoma, in the form of 60-min IV infusions at a dose of 120 mg (m^2^)^−1^ body surface each, on two consecutive days. The representative LC chromatograms of the plasma and urine extracts obtained from the patient after the first IV BM infusion are shown in Fig. 4a–c, respectively. The calculated profile of the plasma concentration–time for BM is presented in Fig. 5a, while the urinary concentrations of BM collected during and after the IV drug infusion are shown in Fig. 5b, respectively. Thus, according to the plasma profile of BM, *C*_max_ at the level of 7015.3 ± 34.5 and 7245.4 ± 42.8 ng mL^−1^ for BM was observed at time of the first and the second IV infusion (*T*_max_ = 1 h) (Fig. 5a). This BM profile obtained in our patient is comparable to the profiles previously reported for pediatric patients after an IV infusion of BM at the dose of 120 mg (m^2^)^−1^ [24, 25]. Urinary concentrations of BM were significantly lower than those observed in plasma (Fig. 5b), what confirms that this drug undergoes extensive metabolism. Specifically, the BM concentrations of 11.3 and 7.3 ng mL^−1^ were found in 24-h urine samples after the first and the second IV BM infusion, respectively. The results stay in line with the previously reported literature data [23–25, 27–29].

## Conclusions

In brief, fast, precise, and accurate LC-FL methods for quantification of BM in human plasma and urine, where cinoxacin was used as the internal standard, were developed and validated. As concerns plasma samples, the sample preparation procedure involved the application of the SPE with C18 cartridges, while a one-step LLE with dichloromethane was applied for the extraction of BM from urine. In both the cases, the LC analysis of BM was carried out on the Synergi Max-RP in the isocratic mode. The proposed methods are time-saving and economical; furthermore, they are suitable for analyzing large numbers of plasma and urine samples for drug monitoring, pharmacokinetic, and biomedical investigations. Finally, the present LC-FL techniques have been successfully used for the monitoring of BM after an IV infusion of BM to a child with refractory progressive Hodgkin’s lymphoma.

## Electronic supplementary material

Below is the link to the electronic supplementary material.

Supplementary material 1 (PDF 309 kb)
