# Supplementary material for: Determination of Bendamustine in Human Plasma and Urine by LC-FL Methods: Application in a Drug Monitoring
Source: Chromatographia. 2016 May 18;79:861–73. doi: 10.1007/s10337-016-3103-3 (PMC4930483; doi:10.1007/s10337-016-3103-3)
Supplement: Supplementary file 1 — Supplementary material 1 (PDF 309 kb) [file 10337_2016_3103_MOESM1_ESM.pdf]

Electronic Supplementary Material

Determination of Bendamustine in Human Plasma and Urine by LC-FL Methods: Application in a Drug Monitoring

Alina Plenis[1]*, Agnieszka Frolow[1], Natalia Rekowska[1], Ilona Olędzka[1], Piotr Kowalski[1], Ewa Bień[2], Małgorzata Anna Krawczyk[2], Elżbieta Adamkiewicz-Drożynska[2], Tomasz Bączek[1]

[1]*Department of Pharmaceutical Chemistry, Medical University of Gdansk, Hallera 107, 80-416 Gdansk, Poland*

[2]*Department of Pediatris, Hematology and Oncology; Medical University Gdansk, Debinki 7, 80-211 Gdansk, Poland*

** Corresponding author; Phone +48 58 3491236; Fax +48 58 3491635;E-mail: aplenis@gumed.edu.pl* (A. Plenis)

Table S1 Analytical extraction test of BM after various extraction procedures for plasma and urine samples (mean ± SD) (n = 3)

Nominal concentration (ng/mL^{-1})	Extraction recovery (%)	Overall Recovery (%)	Extraction recovery (%)	Overall Recovery (%)	Extraction recovery (%)	Overall Recovery (%)
Plasma						
LLE	*tert*-Butyl methyl ether		Ethyl acetate		Dichloromethane	
100	44.6 ± 7.6	43.3 ± 5.9	54.1 ± 6.1	55.5 ± 7.2	75.1 ± 4.2	76.6 ± 5.4
1000	42.1 ± 4.2		56.9 ± 8.3		78.2 ± 6.6	
SPE using Lichrolut RP-18 cartridges and the elution solvent:						
	Acetonitrile		Methanol		Dichloromethane	
100	92.3 ± 6.5	91.2 ± 5.4	99.7 ± 3.8	99.8 ± 3.5	94.9 ± 7.6	94.0 ± 5.4
1000	90.2 ± 4.4		99.9 ± 3.9		93.1 ± 3.3	
SPE using HyperSep™C8 cartridges and the elution solvent:						
	Acetonitrile		Methanol		Dichloromethane	
100	83.7 ± 4.7	83.3 ± 5.2	86.1 ± 7.6	85.9 ± 7.1	82.7 ± 8.6	83.1 ± 8.4
1000	81.9 ± 9.1		85.8 ± 6.6		83.5 ± 8.2	
SPE using Supel-Select HLB cartridges and the elution solvent:						
	Acetonitrile		Methanol		Dichloromethane	
100	85.8 ± 7.7	86.2 ± 6.6	89.3 ± 6.1	88.3 ± 4.7	84.8 ± 7.6	85.4 ± 6.4
1000	86.6 ± 5.5		87.3 ± 3.4		86.1 ± 5.2	
Urine						
LLE	*tert*-Butyl methyl ether		Ethyl acetate		Dichloromethane	
100	62.1 ± 7.7	64.4 ± 8.0	71.7 ± 9.6	70.8 ± 8.4	98.0 ± 5.1	97.8 ± 3.7
1000	66.8 ± 8.4		69.9 ±7.3		97.7 ± 3.1	

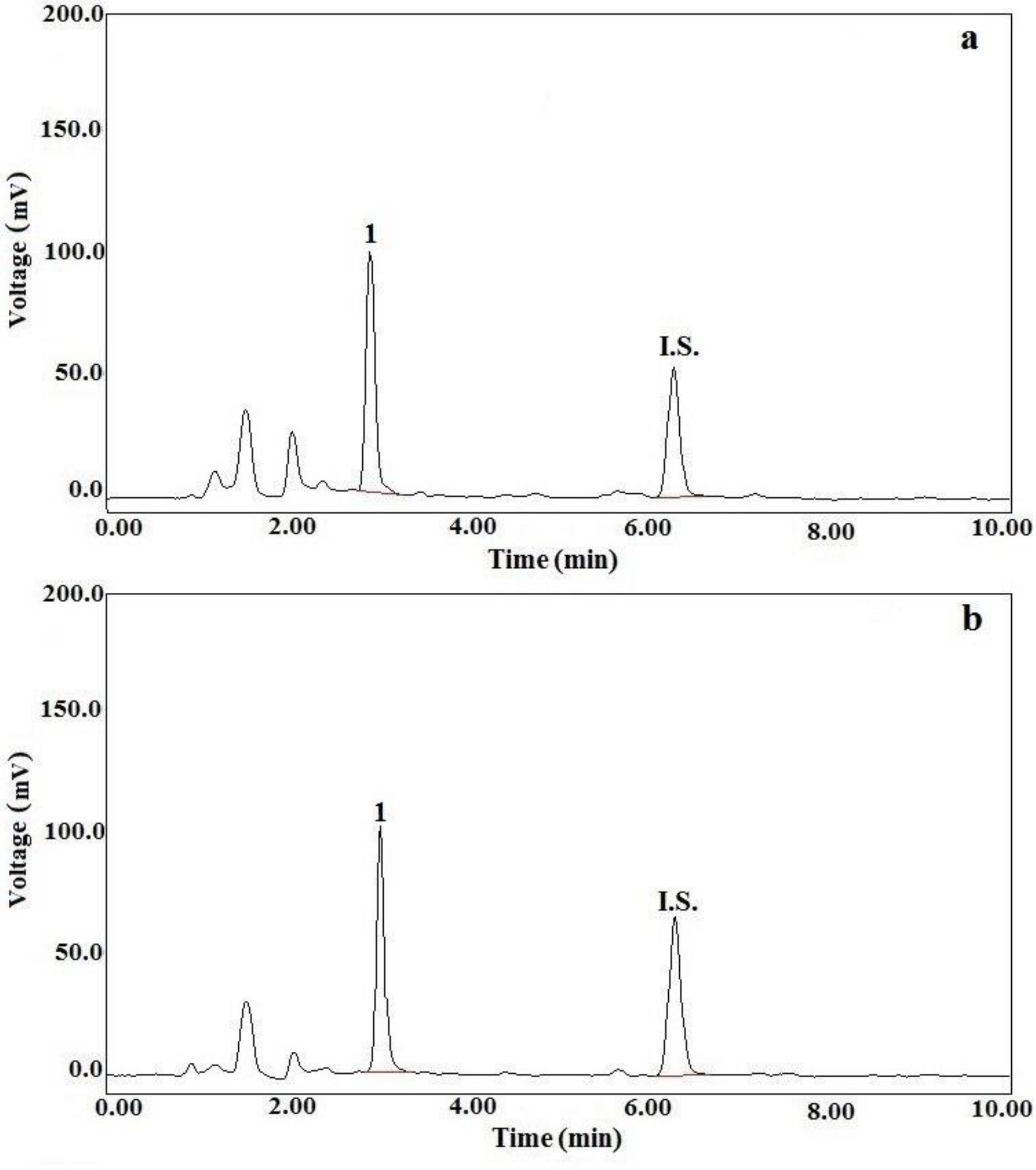

Fig. S1 Representative chromatograms of plasma spiked with 500 ng mL^{-1} of BM and with the I.S. at the level of 1500 ng mL^{-1} obtained in short-term stability test (**a**) and long-term stability test (**b**), respectively

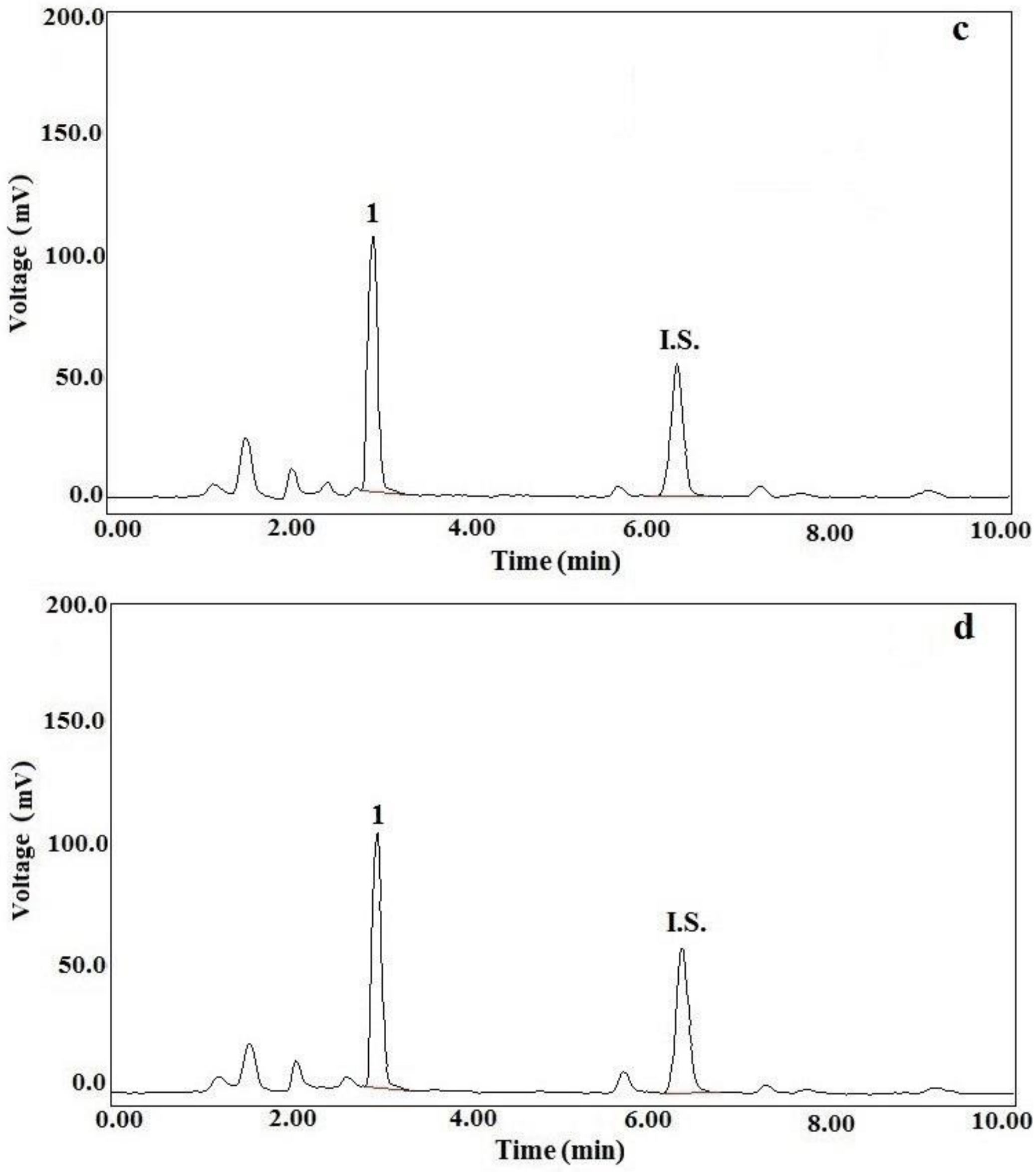

Fig. S2 Representative chromatograms of plasma spiked with 500 ng mL^{-1} of BM and with the I.S. at the level of 1500 ng mL^{-1} performed in freeze/thaw stability test (**c**) and post-preparative storage test (**d**), respectively

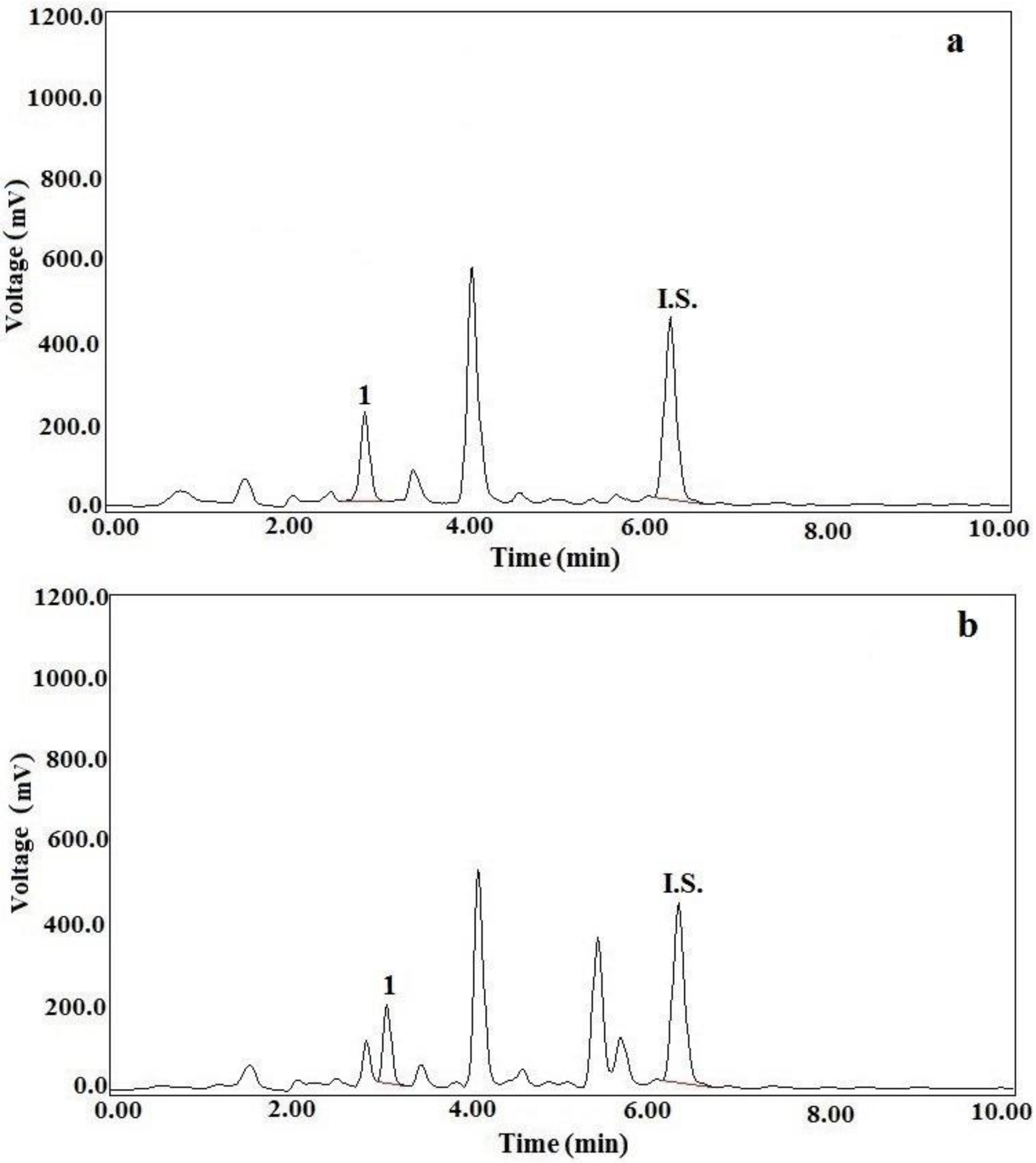

Fig. S3 Typical chromatograms of urine spiked with 250 ng mL^{-1} of BM and with the I.S. at the concentration of 1500 ng mL^{-1} obtained in short-term stability test (**a**) and long-term stability test (**b**), respectively

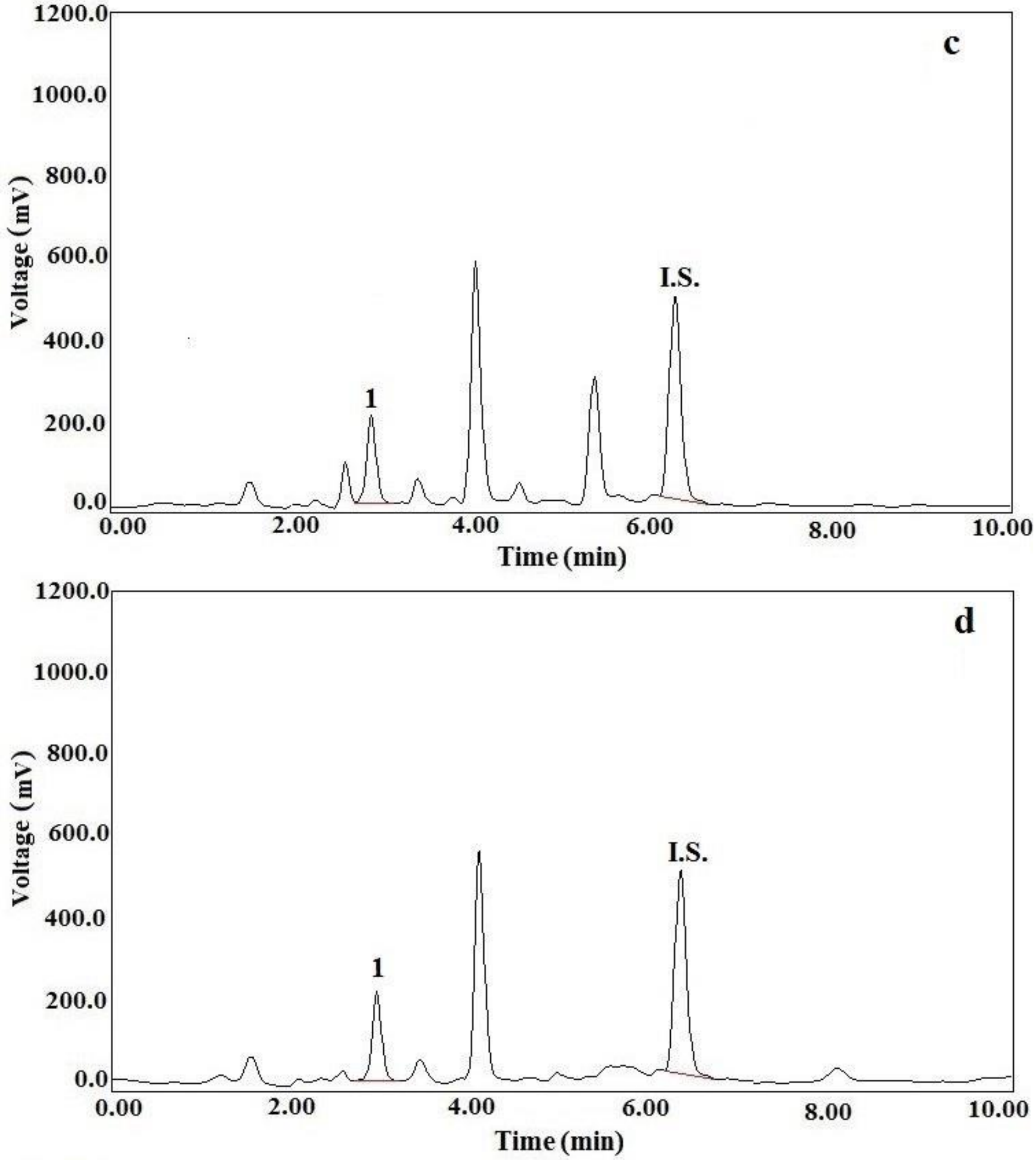

Fig. S4 Typical chromatograms of urine spiked with 250 ng mL^{-1} of BM and with the I.S. at the concentration of 1500 ng mL^{-1} performed in freeze/thaw stability test (**c**) and post-preparative storage test (**d**), respectively
